# Supplementary material for: Optical Analysis of Perovskite III-V Nanowires Interpenetrated Tandem Solar Cells
Source: Nanomaterials (Basel). 2024 Mar 14;14(6):518. doi: 10.3390/nano14060518 (PMC10974091; doi:10.3390/nano14060518)
Supplement: Supplementary file 1 [file nanomaterials-14-00518-s001.zip › nanomaterials-2908294-supplementary.pdf]

Supplementary Materials: Optical Analysis of Perovskite III-V Nanowires Interpenetrated Tandem Solar Cells

Matteo Tirrito [1], Phillip Manley [2], Christiane Becker [3], Eva Unger [3] and Magnus T. Borgström [1,*]

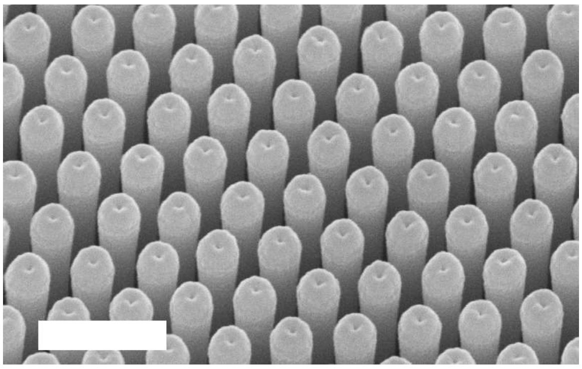

Figure S1. SEM image of NWs embedded in BCB. Scale bar is $1\mu m$

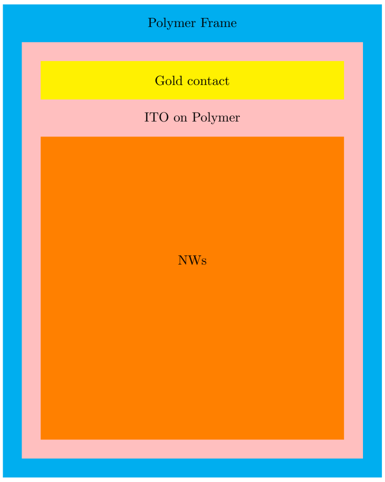

Figure S2. Top view schematic of the 2T tandem solar cell

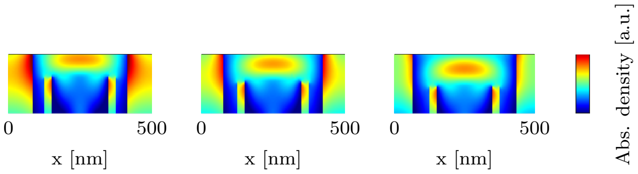

Figure S3. Field absorption density at λ = 490 nm. ITO thickness is 80 nm, 100 nm and 120 nm.

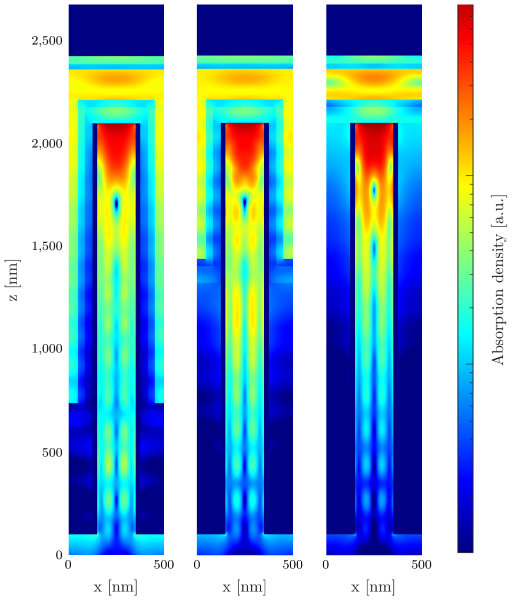

Figure S4. Field absorption density in the structure at λ = 500 nm for 3 terminal solar cell. BCB fill height, from left to right: 500 nm, 1200 nm and planarized. The perovskite overstanding layer is 150 nm thick.

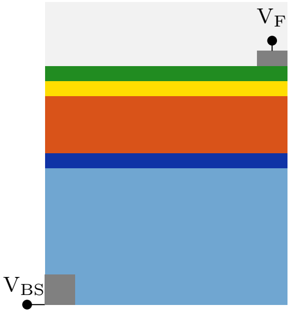

Figure S5. Schematic of the planar material stack. The metal contacts indicate the connection scheme, albeit they are not included in the modelled structure.

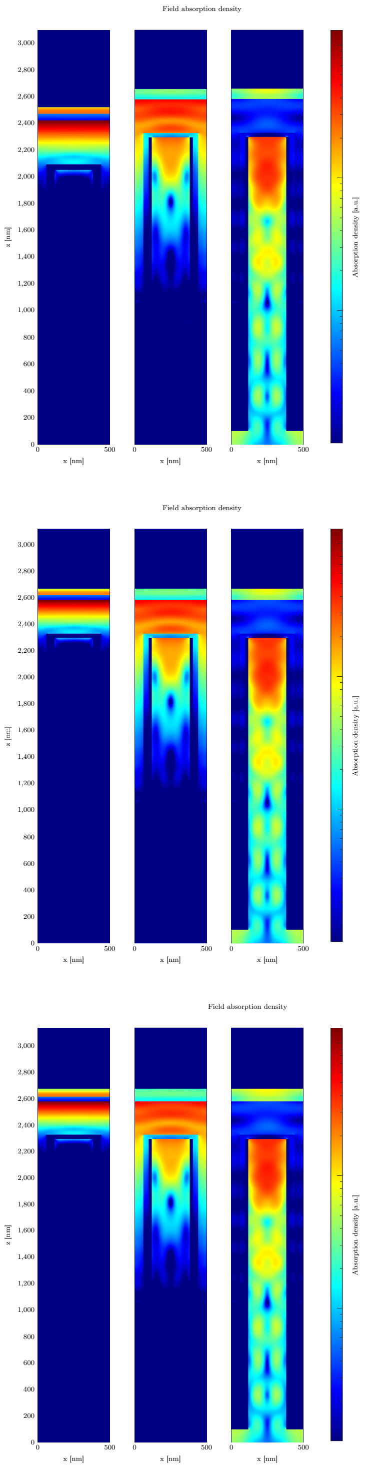

Figure S6. Field absorption density in the structure for different wavelength and different ITO thicknesses. From left to right λ= 400 nm, 600 nm, 800 nm. From top to bottom ITO thickness: 80 nm, 100 nm, 120 nm

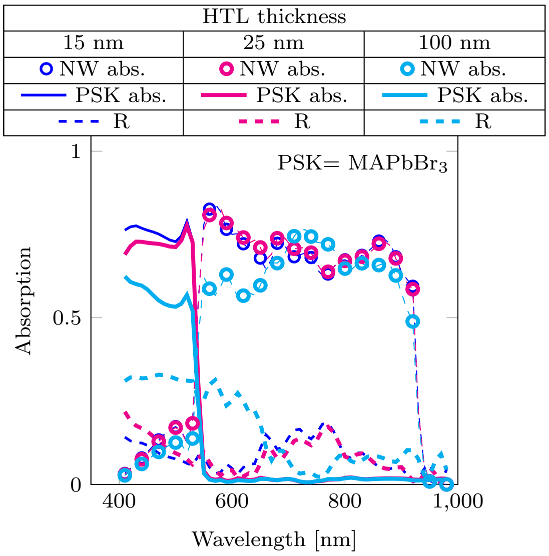

Figure S7. HTL thickness: 15 nm in blue, 25 nm in magenta, 100 nm in cyan.
